# A fully automated sample-to-answer PCR system for easy and sensitive detection of dengue virus in human serum and mosquitos

**DOI:** 10.1371/journal.pone.0218139

**Published:** 2019-07-10

**Authors:** Jih-Jin Tsai, Wei-Liang Liu, Ping-Chang Lin, Bo-Yi Huang, Ching-Yi Tsai, Pei-Yu Alison Lee, Yun-Long Tsai, Pin-Hsing Chou, Simon Chung, Li-Teh Liu, Chun-Hong Chen

**Affiliations:** 1 Center for Dengue Fever Control and Research, Kaohsiung Medical University, Kaohsiung, Taiwan; 2 School of Medicine, College of Medicine, Kaohsiung Medical University, Kaohsiung, Taiwan; 3 Tropical Medicine Center, Kaohsiung Medical University Hospital, Kaohsiung, Taiwan; 4 Division of Infectious Diseases, Department of Internal Medicine, Kaohsing Medical University, Kaohsiung, Taiwan; 5 National Mosquito-Borne Diseases Control Research Center, National Health Research Institutes, Zhunan, Taiwan; 6 GeneReach Biotechnology, Taichung, Taiwan; 7 Department of Medical Laboratory Science and Biotechnology, College of Medical Technology, Chung-Hwa University of Medical Technology, Tainan City, Taiwan; 8 National Institute of Infectious Diseases and Vaccinology, National Health Research Institutes, Zhunan, Taiwan; National Yang-Ming University, TAIWAN

## Abstract

**Background:**

The insulated isothermal PCR (iiPCR) technology enables consistent PCR amplification and detection in a simple heating device. A pan-dengue virus (DENV) RT-iiPCR, targeting the 5’ untranslated region, was validated previously on the semi-automated POCKIT combo system (involving separate devices for nucleic acid extraction and PCR amplification/detection) to offer performance comparable to a laboratory real-time PCR. Working on the same technologies, a compact automated sample-in-answer-out system (POCKIT Central Nucleic Acid Analyser) has been available commercially for iiPCR, minimizing human error risks and allowing easy molecular bio-detection near points of need. Here, we evaluated the analytical and clinical performance of the pan-DENV RT-iiPCR on the fully automated system by comparison to those on the semi-automated system.

**Methodology/Principal findings:**

Testing sera containing serial diluted DENV-1, -2, -3, or -4 cell culture stock, the pan-DENV RT-iiPCR system had similar 100% detection endpoints on the two systems; i.e. at 1, 10, 1 and 10 PFU/ml, respectively, on the fully automated system, and at 10, 1, 10 and 10 PFU/ml, respectively, on the semi-automated system. Furthermore, both fully automated and semi-automated PCR system can detect all four DENV serotypes in mosquitos. Clinical performance of the reagent on the two systems was evaluated by testing 60 human serum samples. Both systems detected the same 40 samples (ten DENV-1, -2, -3, and -4 positive each) and did not detect the other 20; 100% agreement (κ = 1) was found between the two systems.

**Conclusions/Significance:**

With performance comparable to a previously validated system, the fully-automated PCR system allows applications of the pan-DENV reagent as a useful tool near points of need to facilitate easy, fast and effective detection of dengue virus and help mitigate versatile public health challenges in the control and management of dengue disease.

## Introduction

Dengue virus (DENV), a member of the genus Flavivirus in the family Flaviviridae, causes dengue in humans. Dengue virus infection, one of the most important and mosquito-borne viral diseases, is considered a major public health problem in developing tropical and sub-tropical countries mainly in Asia, South America, and the Caribbean with major disease outbreaks and endemic every year [1]. Four serotypes of dengue virus (DENV-1, -2, -3, and -4) have been identified, with distinct genotypes within each serotype. Multiple virus serotypes have been found co-circulating in the hyperendemic regions in Southeast Asia and Pacific [1, 2]. Its single-stranded, positive-sense genomic RNA contains 5'- and 3'- untranslated regions (UTR) and a single open reading frame encoding for a single polyprotein that could be cleaved into three structural proteins (capsid [C], premembrane/membrane [prM/M], and envelope [E]) and seven nonstructural proteins (NS1, NS2A, NS2B, NS3, NS4A, NS4B, and NS5).

DENV is primarily transmitted by the mosquito species *Aedes aegypti* present in tropical and sub-tropical regions, and less efficiently by *A*. *albopictus* [3], particularly during the viremic phase of the disease in which high levels of DENV viremia are present in the blood stream. Positive association between DENV infection in mosquitoes and human has been found; human cases were reported at about one week after appearance of DENV-positive *A*. *aegypti* [4, 5]. *A*. *aegypti* can also pick up DENV from people showing no symptoms or oligo symptom, resulting in silent transmission [6]. Therefore, for dengue disease control, it is important to extend DENV surveillance in the human and mosquito populations to near points of care (POC)/point of needs (PON), generating early alert signals to prevent and/or control DENV epidemics and reducing the spread of DENV infection [7].

DENV causes different clinical signs in humans, from mild febrile illness (dengue fever, DF) to life-threatening condition such as hemorrhagic fever/dengue shock syndrome (DHF/DSS) [8, 9]. Early, accurate and rapid diagnosis of dengue is critical for confirmation of clinically suspected cases to ensure timely management of severe dengue disease [10]. A number of NS1 rapid diagnostic tests are commercially available to easily detect NS1 antigen, but they are not sensitive or specific enough [11–13]. During the acute phase of the illness when viremia levels are high, the viral RNA or soluble antigens can be easily detected [14]. For rapid and accurate diagnosis of dengue virus in early phases of acute infections, detection of DENV RNA is recommended as the most sensitive and specific method to diagnose dengue in the acute phase of the illness by WHO [9]. After the first developed nucleic acid detection test for dengue diagnosis over 20 years ago [15], there have been a number of RT-PCR assays for DENV detection and serotype identification with high sensitivity and specificity [16–20]. Combined with automated RNA extraction protocols, RT-PCR can potentially facilitate fast and accurate diagnosis of dengue suspected cases with a single sample obtained during the patient’s first visit to the clinician. However, the requirement of a pre-PCR nucleic acids extraction step, an expensive and not easy to use thermal cycler, and a highly trained technician make PCR technology not accessible in resource-limited regions where dengue is endemic [10, 21]. Molecular diagnosis tools for dengue is still much needed at or near POC/PON, particularly for developing countries with limited public health resources.

For POC/PON dengue diagnosis, several isothermal amplification methods such as nucleic acid sequence-based amplification (NASBA), thermophilic helicase-dependent amplification (tHDA), loop-mediated isothermal amplification (LAMP), recombinase polymerase amplification (RPA), and insulated isothermal PCR (iiPCR) have been developed and reported previously as potential field-deployable tools for the detection of different microorganisms [7, 10, 22–25]. Based on these technologies, various all-in-one molecular diagnostics have been made available to help minimizing risks of human errors and allowing easy molecular bio-detection at or near POC/PON in the last few years, but, to our knowledge, no reagents are available for DENV testing on these platforms.

The pan-DENV reverse transcription-iiPCR (RT-iiPCR), previously validated to work with the filed-deployable semi-automated PCR system, POCKIT combo (GeneReach Biotech, Taichung, Taiwan), is a potential POC/PON tool for the detection of DENV. It offered great analytical and clinical performances for rapid detection of DENV in human serum; its clinical performance was comparable to that of the reference multiplex RT-PCR assay in studies testing samples collected in Sri Lanka and Taiwan [7, 26]. The field-deployable system includes one automated NA extraction device (taco mini Automatic Nucleic Acid Extraction System, taco mini; GeneReach) and one compact PCR device (POCKIT Nucleic Acid Analyzer, POCKIT; GeneReach) in a durable suitcase for easy field deployment. The iiPCR system is unique in its employment of a single heating source in a thermally baffled device to drive Rayleigh-Bernard convection in a capillary tube, allowing the three steps of PCR (denaturation, annealing, and extension) to occur sequentially and continuously [27, 28]. Since the first report of the assay in 2012, many PCR/RT-iiPCRs on the POCKIT system have been validated for sensitive and specific detection of various parasitic, bacterial, and viral pathogens in animals and humans, including *Plasmodium* spp, *Salmonella* spp, influenza A virus, Middle East respiratory syndrome virus, Zika virus and dengue virus [7, 26, 29–32].

Requiring several manual liquid transfer steps to assemble the PCR tests, the complexity of the semi-automated PCR system is still relatively high. A compact fully automated sample-to-answer system (POCKIT Central Nucleic Acid Analyser, POCKIT Central) is commercially available recently, in which modules in the existing semi-automated system (automated NA extraction and compact POCKIT PCR modules) were integrated with a liquid handling module. This device can be set up and run with minimal set-up time and steps to allow quick and easy detection of DENV in human at settings such as a local hospital laboratory or airport and in mosquitos for surveillance and monitoring of DENV. Therefore, the new assay has potential to enable timely early diagnosis of DENV infection, facilitating effective disease management and control particularly in regions of low medical resources. In addition, the system can facilitate monitoring of the mosquitos carrying DENV in the field to provide timely information for local government to take immediate measures to facilitate effective local management and control of dengue.

In this study, we evaluated the performance of the pan-DENV RT-iiPCR reagent on the POCKIT Central for sensitive and specific detection of DENV1-4 serotypes in human serums and mosquitos.

## Materials and methods

### Ethics statement

Serum samples were collected from clinically suspected dengue patients for routine diagnosis using RT-PCR methods [33] at the Tropical Medicine Center, Kaohsiung Medical University Hospital, Kaohsiung, Taiwan in 2012. The use of retrospective clinical specimens in this study was approved by the Kaohsiung Medical University Hospital Institutional Review Board (KMUHIRB-F(I)-20180032); waiver of informed consent was obtained. All collected samples were anonymized.

### Cells and viruses

Stocks of four DENV isolates, DENV-1 (Hawaii Strain; 1.8 x 10^3^ PFU/mL), DENV-2 (NGC Strain; 2.075 x 10^6^ PFU/mL), DENV-3 (DN8700829A Strain; 1 x 10^6^ PFU/mL), and DENV-4 (DN9000475A Strain; 1.9 x 10^5^ PFU/mL), were prepared in the mosquito C6/36 cell line (*A*. *albopictus*). The virus was diluted in RPMI1640 medium (Gibco-Life Technologies, Grand Island, NY, USA) containing 1% FCS (Gibco-Life Technologies) and added to the cell at a multiplicity of infection of 0.01. After incubation at 28°C, 5% CO_2_ for 4–7 days. Viruses were harvested when cytopathic effect was observed. Titers of DENV stocks and human serum of dengue confirmed cases were determined by plaque assay. Briefly, 10-fold serial dilutions of the DENV stock were made in MEM medium (Gibco-Life Technologies) and added in duplicate to BHK-21 cells in 6-well plates (about 1× 10^6^ cells per well). MEM medium was used in mock infection. Adhesion was allowed at 37°C under 5% CO_2_ for 2 h before addition of 3 mL overlay medium containing 1.2% methylcellulose. Cells were incubated further for 5–10 days until plaques became visually apparent by microscopy, fixed, and stained with crystal violet. Plaques were counted manually and plaque forming units (PFU) per mL were determined with the plaque quantification program [34, 35].

Two viruses known to also cause febrile illness or skin rash illness were tested for analytical specificity. Zika virus (MR766, PRVABC59 strain) were from the American Type Culture Collection, Manassas, VA, USA. Chikungunya virus (CK9500004 strain) was from the Taiwan Center of Disease Control, Taipei, Taiwan.

### Clinical samples

A total of 60 archived serum specimens (30 DENV positive, 10 of DENV-1, -2 and -3 each; 30 DENV negative) were collected from clinically suspected dengue patients with informed consents at the Tropical Medicine Center, Kaohsiung Medical University Hospital, Kaohsiung, Taiwan, for routine diagnosis in 2012. All samples were stored at -80°C until nucleic acid extraction. Due to the lack of DENV-4 271 positive clinical samples in the region, DENV-4 samples were prepared by spiking 10 of the DENV-negative human serum specimens with different concentrations of the DENV-4 DN9000475A stock (1.9 x 10^5^ PFU/mL).

### Mosquito specimens

Adult female mosquitoes, aged 7–8 days, were cold anesthetized and inoculated using a microcapillary needle that had been pulled to a point with needle puller. The 4 serotype of dengue virus stocks were standardized to 2 x 10^6^ PFU/mL, and 0.2 μL was injected into each mosquito (approximately 400 PFU/mosquito). Infected mosquitoes were maintained in cages at 28 ± 1°C and 70% ± 5% relative humidity with a 12 h/12 h light-dark cycle and fed with 10% sucrose solution. *A*. *aegypti* mosquitoes (UGAL strain) were injected individually with DENV-1 (Hawaii Strain, 400 PFU), DENV-2 (NGC Strain, 400 PFU), DENV-3 (DN8700829A Strain, 400 PFU), or DENV-4 (DN9000475A Strain, 400 PFU) by micro-injection (nanoinjector) into the thoracic cavity. Whole mosquitoes were homogenized for plaque forming assay after 0, 1, 3, 5, 7, and 14 days post-infection [36]. DENV was found to be detectable (10^3^–10^4^ PFU/ml) in mosquitoes, and viral titers (infectivity) were maintained in the mosquitoes that had been cultured for 2 weeks without significant attenuation. Three infected mosquitoes were collected on day 7 post infection, and frozen at −80°C until further use. For nucleic acid extraction before PCR analysis, each mosquito was homogenized in 250 μl PBS with a disposable grinder and centrifuged briefly. Subsequently, 200 μl of the upper aqueous sample were transferred into the first well of the preloaded extraction plate or cartridge.

### Pan-DENV RT-iiPCR Assay on POCKIT combo

The pan-DENV RT-iiPCR reagent, targeting the 5’ UTR sequence, was performed as described in the user manual (POCKIT Dengue Virus Reagent Set, GeneReach Biotech). The semi-automated POCKIT combo system includes an automated taco mini for nucleic acid extraction and a POCKIT device for PCR detection. Nucleic acid extraction on the taco mini was done using the taco Preloaded DNA/RNA Extraction Kit (GeneReach Biotech) according to the manufacturer’s instructions. Briefly, 200 μL of the sample were added into the first well of the extraction plate before the automatic extraction steps. All nucleic acids were collected individually and placed at -80°C until further use. Subsequent PCR detection was done manually on a POCKIT device. First, 50 μL of Premix Buffer was added to reconstitute each lyophilized Dengue Virus Premix, followed by the addition of 5 μL of test nucleic acid extract. A 50 μL volume of the premix/sample mixture was transferred into an R-tube, which was sealed subsequently with a cap, spun briefly in a microcentrifuge (cubee, GeneReach Biotech), and placed into a POCKIT device. The default program, including an RT step at 50°C for 10 min and an iiPCR step at 95°C for about 30 min; qualitative results were shown on the display screen in less than one hour.

### Pan-DENV RT-iiPCR assay on POCKIT central

To run the pan-DENV RT-iiPCR reagent (POCKIT Dengue Virus Reagent Set) on the fully automated sample-to-answer POCKIT Central system, the premix tube of the reagent was placed into the designated well in the Transfer Cartridge of the POCKIT Cartridge Set (GeneReach Biotech). After 200 μL of the clinical sample were added into well one of the Extraction Cartridge of the POCKIT Cartridge Set, and sample and reagent information were logged into the device, the cartridges were placed into the POCKIT Central device accordingly, and the program was started. The nucleic acid extraction, iiPCR amplification and detection steps were completed automatically in 80 minutes. Qualitative results were displayed on the screen at the end of the program.

### Statistical analysis

To compare the performance of two methods, inter-rater agreement was assessed with a 2 x 2 contingency table and determined by Cohen’s kappa statistic using commercial software SPSS 14.0 (SPSS Inc., Chicago, IL). The values ≤ 0.20, 0.21–0.39, 0.40–0.59, 0.60–0.79, 0.80–0.90, and > 0.90 were interpreted as no, minimal, weak, moderate, strong, and almost perfect agreement, respectively [37].

## Results

### Analytical sensitivity

To test analytical sensitivity, 10-fold serial dilutions (1,000, 100, 10, 1, 0.1, and 0.01 PFU/mL) of the DENV-1, 2, 3, or 4 isolates were made separately in pooled DENV-negative human serum and subjected in triplicate to the pan-DENV RT-iiPCR test with the semi-automated POCKIT combo and automated POCKIT Central systems in parallel. The 100% detection end points were at 1, 10, 1, and 10 PFU/mL for DENV-1, -2, -3 and -4 with the fully automated PCR system, respectively; while the end points were at 10, 1, 10, and 10 PFU/mL for DENV-1, -2, -3 and -4 with the semi-automated PCR system, respectively (Table 1, S1 Table). The results indicated that performances of the pan-DENV RT-iiPCR on the two PCR systems were comparable.

**Table 1:** Analytical sensitivity of pan-DENV RT-iiPCR on the fully automated POCKIT Central system: Comparison with the semi-automated POCKIT combo system.

Pathogen	PUF/mL	POCKIT combo	POCKIT Central
**DENV-1**	**10**^**3**^	3/3	3/3
	**10**^**2**^	3/3	3/3
	**10**^**1**^	3/3	3/3
	**10**^**−0**^	0/3	3/3
	**10**^**−1**^	0/3	0/3
**DENV-2**	**10**^**3**^	3/3	3/3
	**10**^**2**^	3/3	3/3
	**10**^**1**^	3/3	3/3
	**10**^**−0**^	3/3	2/3
	**10**^**−1**^ **10**^**−2**^	0/3 0/3	1/3 0/3
**DENV-3**	**10**^**3**^	3/3	3/3
	**10**^**2**^	3/3	3/3
	**10**^**1**^	3/3	3/3
	**10**^**−0**^	2/3	3/3
	**10**^**−1**^	0/3	1/3
	**10**^**−2**^	0/3	0/3
**DENV-4**	**10**^**3**^	3/3	3/3
	**10**^**2**^	3/3	3/3
	**10**^**1**^	3/3	3/3
	**10**^**−0**^	1/3	2/3
	**10**^**−1**^	0/3	0/3

DENV, dengue virus; PFU, plaque forming unit; RT-iiPCR, reverse transcription-insulated isothermal PCR.

### Analytical specificity

Similar to the observation with the semi-automated PCR system, the pan-DENV RT-iiPCR with the automated PCR system detected the four DENV serotypes (DENV-1, -2, -3, and -4) and did not react with the two Zika virus and one chikungunya virus strains, indicating that the assay also had great specificity for DENV RNA on the fully automated system (Table 2).

**Table 2:** Analytical specificity of pan-DENV RT-iiPCR on the fully automated POCKIT Central system comparison with the semi-automated POCKIT combo system.

Pathogen	pan-DENV RT-iiPCR	
	POCKIT combo	POCKIT Central
Dengue virus serotype 1	+	+
Dengue virus serotype 2	+	+
Dengue virus serotype 3	+	+
Dengue virus serotype 4	+	+
Zika virus (MR766 strain)	-	-
Zika virus (PRVABC59 strain)	-	-
Chikungunya virus (CK9500004 strain)	-	-

RT-iiPCR, reverse transcription-insulated isothermal PCR.

### Clinical performance

Performance of the pan-DENV RT-iiPCR on the semi-automated POCKIT combo system (see Materials and Methods for detail) was previously validated to be equivalent to that of the CDC DENV-1-4 real-time RT-PCR [7]. The clinical performance of the pan-DENV RT-iiPCR reagent on the fully automated POCKIT Central system was evaluated by comparison with the performance on the semi-automated PCR system. For this purpose, clinical serum samples were tested from sample to results using the pan-DENV RT-iiPCR reagent on the fully automated and semi-automated PCR systems in parallel. A total of 60 archived serum specimens (30 DENV positive, 10 of DENV-1, -2 and -3 each; 30 DENV negative) collected from clinically suspected dengue patients were tested. There were no severe dengue patients in the 30 DENV-positive group. The 30 control cases were selected from age- and sex-matched dengue-suspected cases; dengue virus infections were later ruled out clinically and by laboratory tests. All clinical samples were collected within 6 days from the day of illness onset. Based on analysis from a previous study, the median age was 45 years old (range 20–65) in the case group and 46 years old (range 21–67) in the control group. The percentage of male subjects was 53.3% in the case group and 50% in the control group. The median viral titers in different serotypes were 8.2 x 10^2^ PFU/mL (DENV-1), 3.5 x 10^3^ PFU/mL (DENV-2) and 2.7 x 10^5^ PFU/mL (DENV-3) (S2 Table). DENV-4 samples were prepared by spiking 10 of the DENV-negative human serum specimens with different concentrations of the DENV-4 DN9000475A stock (1.9 x 10^5^ PFU/mL). The results showed that the pan-DENV RT-iiPCR detected 40 positive samples and 20 negative samples by both PCR systems (Table 3, S2 Table). The overall agreement between the two systems was 100% (CI_95%_, 95.7–100%; κ = 1.0), indicating the reagent can work with the fully automated PCR system to provide performance equivalent to that with the semi-automated PCR system for the detection of DENV RNA in human serum.

**Table 3:** Clinical performance of pan-DENV RT-iiPCR on fully automated POCKIT Central system: Comparison with semi-automated POCKIT combo system.

POCKIT Central	POCKIT combo		Total (POCKIT Central)
	Positive	Negative	
Positive	40	0	40
Negative	0	20	20
Total (POCKIT combo)	40	60	60
**Total Agreement: 100% (CI**_**95%**_**: 95.68–100%);** κ = 1.0			

### Detection of dengue virus serotypes 1–4 in mosquitos

DENV titers can reach 10^2.67 ± 0.33^ to 10^4.09 ± 0.71^ PFU equivalents/mL in infected female *A*. *aegypti a*fter oral infection with DENV [36]. The titers are higher than the sensitivity of the pan-DENV RT-iiPCR/POCKIT Central system (Table 1). Preliminary evaluation of the performance of the fully automated and semi-automated PCR systems was done using mosquitos collected on day 7 post intra-thoracic infection with one of the four serotypes. The results shows that both PCR systems could detect DENV serotype 1-, 2-, 3-, and 4 RNA in DENV-infected mosquitos (Table 4; each serotype tested in triplicate, S3 Table), suggesting both systems can be a useful tool for screening DENV-infected mosquitos.

**Table 4:** Detection of the four dengue virus serotypes in *Aedes aegypti* by pan-DENV RT-iiPCR on both fully automated and semi-automated POCKIT systems.

POCKIT system	Dengue virus			
	serotype 1	serotype 2	serotype 3	serotype 4
**Fully automated**	+	+	+	+
**Semi-automated**	+	+	+	+

## Discussion

Testing with the pan-DENV RT-iiPCR, the analytical and clinical performance of the fully automatic POCKIT Central system was comparable to those of the semi-automatic POCKI combo system, which was validated previously to offer performances equivalent to the CDC DENV1-4 real-time RT-PCR for the detection of DENV in human serum [7, 24, 26]. Validation with 60 serum samples prepared from dengue-suspected patients demonstrated that the pan-DENV RT-iiPCR performed equally well on both the semi-automated and fully automated equipment (100% agreement; CI_95%_, 98.81 ~ 100%; κ = 1.0; Table 3). Moreover, the pan-DENV RT-iiPCR can work on both systems to detect the four DENV serotypes in DENV-infected mosquitos (Table 4).

Timely on-site detection of DENV in human and mosquito can potentially alert front-line health professionals to allow timely implementation of intervention strategies and help focusing efforts in targeted areas to help mitigate disease outbreaks [1, 38]. Performance of such tests near POC/PON could help shift health service more to local levels, achieve disease diagnosis at early stages, and reduce costs and turn-around time, improving health management quality in under-reached communities. Currently, commercially available NS1 immunological test products are rapid and do not require trained personnel to detect DENV in both human and mosquitos [39]. However, they are reported as less sensitive than ELISA and may cross react with other flaviviruses [11–13]. Moreover, their sensitivities were relatively low on days 1 and 2 and after day 5 post-symptomatic onset in human, compared to that seen with the RT-PCR methods [40].

PCR testing after an NS1 positive results has been recommended for confirmation of DENV infection, but the samples have to be shipped to a central laboratory currently. There are needs for sensitive and specific molecular diagnostic tests for dengue at POC/PON to allow early intervention for clinical management, surveillance, and outbreak investigations [26]. The previously available pan-DENV RT-iiPCR/semi-automated PCR system enabled sensitive and specific molecular testing near POC/PON. In this study, we showed that the reagent also worked well on the fully automated PCR system, which can serve as an advanced simple tool at settings with limited human resources and infrastructure to set up a PCR laboratory.

**Fig 1:**
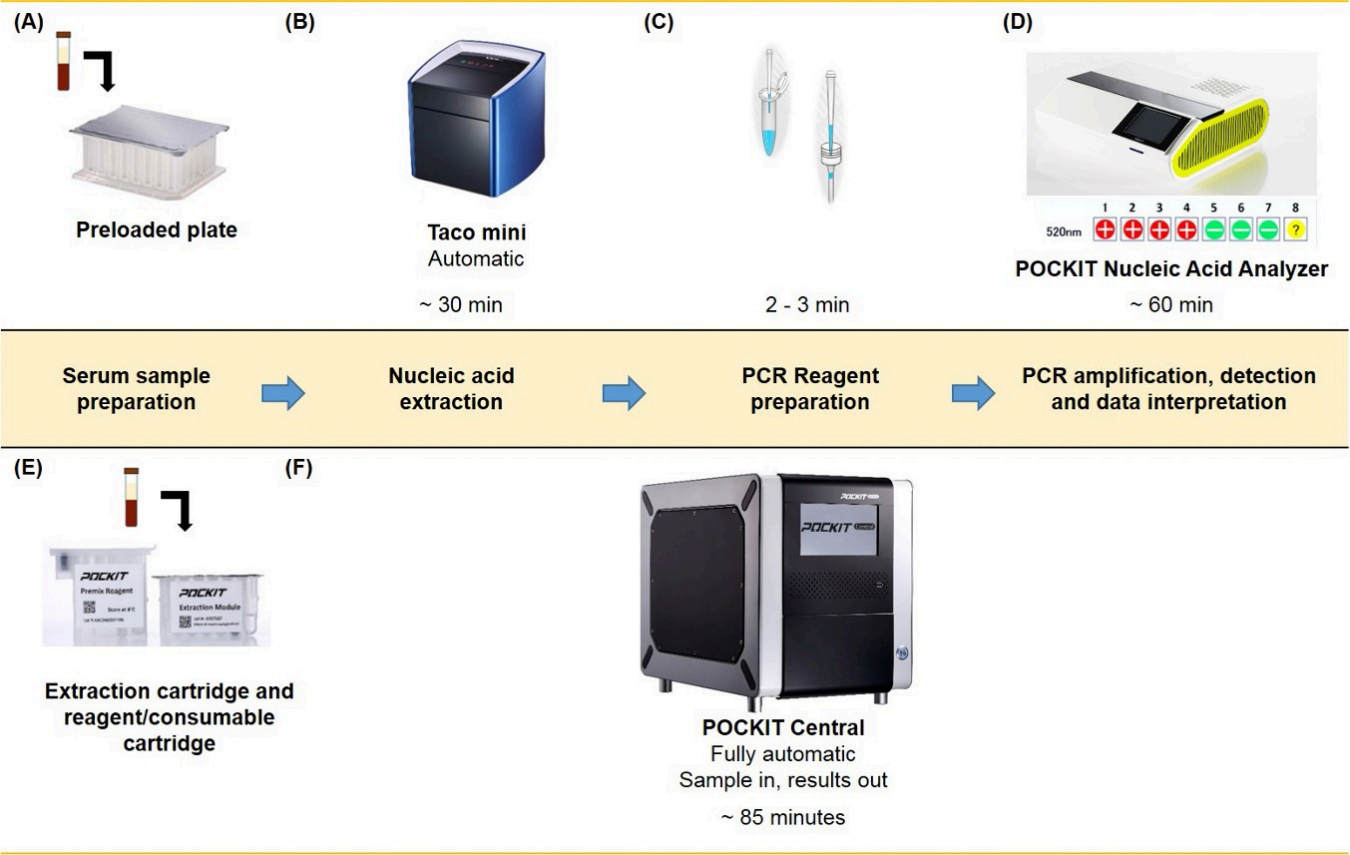
Workflow of the semi-automatic and full-automatic PCR systems. In the semi-automatic system, users add sample (serum, homogenized mosquitos) into the preloaded extraction plate (A) and place the plate into the nucleic acid extraction device for automatic nucleic acid extraction (B). The resulted nucleic acid extracts are added manually to the lyophilized reagent reconstituted first with provided buffer; the final PCR mixture is transferred manually to the reaction vessel (C). The reaction vessels are placed into the PCD device, which performs PCR amplification, detection and data interpretation automatically to provide qualitative results on the monitor (D). In the fully automatic system, users add serum sample to the preloaded extraction cartridge, place the extraction and reagent/consumable cartridges into the device, key in reagent and sample information, and start the assay (E). The device automates nucleic acid extraction, PCR reagent preparation, and PCR amplification, detection, and data interpretation to provide qualitative results on the monitor (F).

One major advantages of the fully automated PCR system is its simple protocol (Fig 1); users simply have to put 200 uL serum into a preloaded extraction cartridge, place both the extraction and reagent cartridges into the device, and start the reaction through the user interface on the screen; the results are ready in 85 min. Compared to conventional PCR methods and to the semi-automated PCR system (see Fig 1 for procedures involved), the fully automated PCR system offers much shorter set up time; its capacity to process 8 reactions in one test run makes it suitable for applications at near-patient settings where the sample numbers are relatively small and a sophisticated testing procedure is not possible. Moreover, the pan-DENV RT-iiPCR reagent is available in a lyophilized format to facilitate shipment at ambient temperatures and storage at 2–8°C for at least 2 years, greatly reducing shipping and storage costs. Hence, this system can facilitate relatively inexpensive, rapid, and simple POC/PON detection of DENV in routine dengue diagnosis.

Moreover, the fully automated and semi-automated PCR systems allows applications of the pan-DENV RT-iiPCR to detect DENV in the local mosquito populations even at remote regions; the combined results can help decision making to effectively allocate control and prevention efforts into areas most in need of dengue control. Therefore, the fully automated POCKIT Central device and field-deployable semi-automated POCKIT combo system has potential to serve as a flexible mobile PON tool for rapid DENV detection in both human and mosquito. Studies to verify and validate further the performance of the pan-DENV RT-iiPCR reagents on both PCR systems for DENV detection in mosquitos are underway.

In summary, the pan-DENV RT-iiPCR coupled with the fully automated and semi-automated POCKIT platforms can serve as a rapid, accurate, and effective tool for use as a POC/PON test system for early differential diagnosis of DENV infection in human especially in local clinics, laboratories and hospitals, or for surveillance of DENV in mosquitos.

## Supporting information

S1 Table Qualitative test results for analytical sensitivity analysis of pan-DENV RT-iiPCR on the fully automated POCKIT Central system and the semi-automated POCKIT combo system. (DOCX)

S2 Table Sample info and test results of clinical and mosquito samples with pan-DENV RT-iiPCR on fully automated POCKIT Central system and semi-automated POCKIT combo system. (DOCX)

S3 Table Sample info and test results of mosquito samples with pan-DENV RT-iiPCR on fully automated POCKIT Central system and semi-automated POCKIT combo system. (DOCX)
