# Correction: A fully automated sample-to-answer PCR system for easy and sensitive detection of dengue virus in human serum and mosquitos

**DOI:** 10.1371/journal.pone.0315117

**Published:** 2024-12-03

**Authors:** The PLOS ONE Staff

The first and last authors, Jih-Jin and Chun-Hong Chen, should not have been attributed equal contribution to this work.

The publisher apologizes for the error.
