# Supplementary material for: A fully automated sample-to-answer PCR system for easy and sensitive detection of dengue virus in human serum and mosquitos
Source: PLoS One. 2019 Jul 10;14(7):e0218139. doi: 10.1371/journal.pone.0218139 (PMC6619671; doi:10.1371/journal.pone.0218139)
Supplement: S1 Table — (DOCX) [file pone.0218139.s001.docx]

# S1 Table Qualitative test results for analytical sensitivity analysis of pan-DENV RT-iiPCR on the fully automated POCKIT Central system and the semi-automated POCKIT combo system

| **Pathogen** | **PUF/mL** | **POCKIT combo** | **POCKIT Central** |
| --- | --- | --- | --- |
| **DENV-1** | **10^3^** | +, +, + | +, +, + |
|  | **10^2^** | +, +, + | +, +, + |
|  | **10^1^** | +, +, + | +, +, + |
|  | **10^-0^** | -, -, - | +, +, + |
|  | **10^-1^** | -, -, - | -, -, - |
| **DENV-2** | **10^3^** | +, +, + | +, +, + |
|  | **10^2^** | +, +, + | +, +, + |
|  | **10^1^** | +, +, + | +, +, + |
|  | **10^-0^** | +, +, + | +, +, - |
|  | **10^-1^**  **10^-2^** | -, -, -  -, -, - | -, -, +  -, -, - |
| **DENV-3** | **10^3^** | +, +, + | +, +, + |
|  | **10^2^** | +, +, + | +, +, + |
|  | **10^1^** | +, +, + | +, +, + |
|  | **10^-0^** | +, +, - | +, +, + |
|  | **10^-1^** | -, -, - | -, -, + |
|  | **10^-2^** | -, -, - | -, -, - |
| **DENV-4** | **10^3^** | +, +, + | +, +, + |
|  | **10^2^** | +, +, + | +, +, + |
|  | **10^1^** | +, +, + | +, +, + |
|  | **10^-0^** | -, -, + | -, +, + |
|  | **10^-1^** | -, -, - | -, -, - |

DENV, dengue virus; PFU, plaque forming unit; RT-iiPCR, reverse transcription-insulated isothermal PCR; POCKIT Central, POCKIT Central Nucleic Acid Analyzer; POCKIT combo, including taco mini Automatic Nucleic Acid Extraction System and POCKIT Nucleic Acid Analyzer.
