# Supplementary material for: A fully automated sample-to-answer PCR system for easy and sensitive detection of dengue virus in human serum and mosquitos
Source: PLoS One. 2019 Jul 10;14(7):e0218139. doi: 10.1371/journal.pone.0218139 (PMC6619671; doi:10.1371/journal.pone.0218139)
Supplement: S2 Table — (DOCX) [file pone.0218139.s002.docx]

**S2 Table** Sample info and test results of clinical samples with pan-DENV RT-iiPCR on fully automated POCKIT Central system and semi-automated POCKIT combo system

| **No.** | **Case/Control** | **Age** | **Gender** | **DENV serotype** | **DENV titer** | **POCKIT Central** | **POCKIT combo** |
| --- | --- | --- | --- | --- | --- | --- | --- |
| H1 | Case | 36 | Female | DENV-1 | 1 x 10^1^ | Positive | positive |
| H2 | Case | 51 | Female | DENV-1 | 6.6 x 10^3^ | Positive | positive |
| H3 | Case | 23 | Male | DENV-1 | 1 x 10^4^ | Positive | positive |
| H4 | Case | 55 | Male | DENV-1 | 2.1 x 10^2^ | Positive | positive |
| H5 | Case | 42 | Female | DENV-1 | 6.9 x 10^2^ | Positive | positive |
| H6 | Case | 27 | Male | DENV-1 | 8.9 x 10^3^ | Positive | Positive |
| H7 | Case | 65 | Male | DENV-1 | 2.7 x 10^3^ | Positive | Positive |
| H8 | Case | 47 | Male | DENV-1 | 3.9 x 10^1^ | Positive | Positive |
| H9 | Case | 31 | Female | DENV-1 | 9.5 x 10^2^ | Positive | Positive |
| H10 | Case | 63 | Female | DENV-1 | 6.7 x 10^1^ | Positive | Positive |
| H11 | Case | 20 | Male | DENV-2 | 1 x 10^2^ | Positive | Positive |
| H12 | Case | 59 | Male | DENV-2 | 1 x 10^5^ | Positive | Positive |
| H13 | Case | 39 | Female | DENV-2 | 6.8 x 10^2^ | Positive | Positive |
| H14 | Case | 25 | Male | DENV-2 | 1.2 x 10^3^ | Positive | Positive |
| H15 | Case | 44 | Male | DENV-2 | 3.2 x 10^3^ | Positive | Positive |
| H16 | Case | 29 | Female | DENV-2 | 7.9 x 10^4^ | Positive | Positive |
| H17 | Case | 46 | Female | DENV-2 | 7.2 x 10^3^ | Positive | Positive |
| H18 | Case | 48 | Male | DENV-2 | 2.2 x 10^4^ | Positive | Positive |
| H19 | Case | 60 | Female | DENV-2 | 3.8 x 10^3^ | Positive | Positive |
| H20 | Case | 62 | Male | DENV-2 | 2.3 x 10^2^ | Positive | Positive |
| H21 | Case | 51 | Male | DENV-3 | 1 x 10^2^ | Positive | Positive |
| H22 | Case | 21 | Male | DENV-3 | 9.7 x 10^5^ | Positive | Positive |
| H23 | Case | 53 | Female | DENV-3 | 2.2 x 10^3^ | Positive | Positive |
| H24 | Case | 24 | Female | DENV-3 | 4.6 x 10^4^ | Positive | Positive |
| H25 | Case | 56 | Male | DENV-3 | 1.2 x 10^5^ | Positive | Positive |
| H26 | Case | 38 | Female | DENV-3 | 1 x 10^6^ | Positive | Positive |
| H27 | Case | 49 | Male | DENV-3 | 5.3 x 10^5^ | Positive | Positive |
| H28 | Case | 50 | Male | DENV-3 | 8.1 x 10^5^ | Positive | Positive |
| H29 | Case | 33 | Female | DENV-3 | 5.8 x 10^2^ | Positive | Positive |
| H30 | Case | 28 | Female | DENV-3 | 4.2 x 10^5^ | Positive | Positive |
| H31 | Control | 67 | Male | - | - | Negative | Negative |
| H32 | Control | 25 | Male | - | - | Negative | Negative |
| H33 | Control | 47 | Female | - | - | Negative | Negative |
| H34 | Control | 50 | Female | - | - | Negative | Negative |
| H35 | Control | 29 | Female | - | - | Negative | Negative |
| H36 | Control | 53 | Male | - | - | Negative | Negative |
| H37 | Control | 32 | Male | - | - | Negative | Negative |
| H38 | Control | 55 | Female | - | - | Negative | Negative |
| H39 | Control | 59 | Male | - | - | Negative | Negative |
| H40 | Control | 61 | Female | - | - | Negative | Negative |
| H41 | Control | 40 | Female | - | - | Negative | Negative |
| H42 | Control | 53 | Male | - | - | Negative | Negative |
| H43 | Control | 42 | Male | - | - | Negative | Negative |
| H44 | Control | 62 | Male | - | - | Negative | Negative |
| H45 | Control | 45 | Male | - | - | Negative | Negative |
| H46 | Control | 26 | Female | - | - | Negative | Negative |
| H47 | Control | 47 | Female | - | - | Negative | Negative |
| H48 | Control | 49 | Female | - | - | Negative | Negative |
| H49 | Control | 27 | Female | - | - | Negative | Negative |
| H50 | Control | 52 | Female | - | - | Negative | Negative |
| H51 | Control | 30 | Male | - | - | NP | NP |
| H52 | Control | 40 | Female | - | - | NP | NP |
| H53 | Control | 54 | Female | - | - | NP | NP |
| H54 | Control | 33 | Male | - | - | NP | NP |
| H55 | Control | 57 | Male | - | - | NP | NP |
| H56 | Control | 35 | Female | - | - | NP | NP |
| H57 | Control | 39 | Female | - | - | NP | NP |
| H58 | Control | 43 | Male | - | - | NP | NP |
| H59 | Control | 63 | Male | - | - | NP | NP |
| H60 | Control | 21 | Male | - | - | NP | NP |
| H51* | Control | 30 | Male | DENV-4 | 9.5 x 10^4^ | Positive | Positive |
| H52* | Control | 40 | Female | DENV-4 | 9.5 x 10^4^ | Positive | Positive |
| H53* | Control | 54 | Female | DENV-4 | 6.3 x 10^4^ | Positive | Positive |
| H54* | Control | 33 | Male | DENV-4 | 6.3 x 10^4^ | Positive | Positive |
| H55* | Control | 57 | Male | DENV-4 | 4.8 x 10^4^ | Positive | Positive |
| H56* | Control | 35 | Female | DENV-4 | 4.8 x 10^4^ | Positive | Positive |
| H57* | Control | 39 | Female | DENV-4 | 4.8 x 10^4^ | Positive | Positive |
| H58* | Control | 43 | Male | DENV-4 | 3.8 x 10^4^ | Positive | Positive |
| H59* | Control | 63 | Male | DENV-4 | 3.8 x 10^4^ | Positive | Positive |
| H60* | Control | 21 | Male | DENV-4 | 3.8 x 10^4^ | Positive | Positive |

DENV, dengue virus; POCKIT Central, POCKIT Central Nucleic Acid Analyzer; POCKIT combo, including taco mini Automatic Nucleic Acid Extraction System and POCKIT Nucleic Acid Analyzer; -, DENV negative, NP, not performed; *, spiked with DENV-4 (DN9000475A Strain)
