# Supplementary material for: A fully automated sample-to-answer PCR system for easy and sensitive detection of dengue virus in human serum and mosquitos
Source: PLoS One. 2019 Jul 10;14(7):e0218139. doi: 10.1371/journal.pone.0218139 (PMC6619671; doi:10.1371/journal.pone.0218139)
Supplement: S3 Table — (DOCX) [file pone.0218139.s003.docx]

**S3 Table** Sample info and test results of mosquito samples with pan-DENV RT-iiPCR on fully automated POCKIT Central system and semi-automated POCKIT combo system

| **No.** | **DENV serotype** | **POCKIT Central** | **POCKIT combo** |
| --- | --- | --- | --- |
| M1 | DENV-1 | Positive | Positive |
| M2 | DENV-1 | Positive | Positive |
| M3 | DENV-1 | Positive | Positive |
| M4 | DENV-2 | Positive | Positive |
| M5 | DENV-2 | Positive | Positive |
| M6 | DENV-2 | Positive | Positive |
| M7 | DENV-3 | Positive | Positive |
| M8 | DENV-3 | Positive | Positive |
| M9 | DENV-3 | Positive | Positive |
| M10 | DENV-4 | Positive | Positive |
| M11 | DENV-4 | Positive | Positive |
| M12 | DENV-4 | Positive | Positive |
| M13 | Negative | Negative | Negative |
| M14 | Negative | Negative | Negative |
| M15 | Negative | Negative | Negative |

DENV, dengue virus; POCKIT Central, POCKIT Central Nucleic Acid Analyzer; POCKIT combo, including taco mini Automatic Nucleic Acid Extraction System and POCKIT Nucleic Acid Analyzer.
